# Application of deep learning technology for temporal analysis of videofluoroscopic swallowing studies

**DOI:** 10.1038/s41598-023-44802-3

**Published:** 2023-10-16

**Authors:** Seong Yun Jeong, Jeong Min Kim, Ji Eun Park, Seung Jun Baek, Seung Nam Yang

**Affiliations:** 1 Department of Computer Science and Engineering, Korea University, 145 Anam-ro Seongbuk-gu, Seoul, 02841 Korea; 2 Department of Physical Medicine and Rehabilitation, Korea University Guro Hospital, Korea University College of Medicine, 148, Gurodong-ro, Guro-gu, Seoul, 08308 Korea

**Keywords:** Medical research, Engineering

## Abstract

Temporal parameters during swallowing are analyzed for objective and quantitative evaluation of videofluoroscopic swallowing studies (VFSS). Manual analysis by clinicians is time-consuming, complicated and prone to human error during interpretation; therefore, automated analysis using deep learning has been attempted. We aimed to develop a model for the automatic measurement of various temporal parameters of swallowing using deep learning. Overall, 547 VFSS video clips were included. Seven temporal parameters were manually measured by two physiatrists as ground-truth data: oral phase duration, pharyngeal delay time, pharyngeal response time, pharyngeal transit time, laryngeal vestibule closure reaction time, laryngeal vestibule closure duration, and upper esophageal sphincter opening duration. ResNet3D was selected as the base model for the deep learning of temporal parameters. The performances of ResNet3D variants were compared with those of the VGG and I3D models used previously. The average accuracy of the proposed ResNet3D variants was from 0.901 to 0.981. The F1 scores and average precision were 0.794 to 0.941 and 0.714 to 0.899, respectively. Compared to the VGG and I3D models, our model achieved the best results in terms of accuracy, F1 score, and average precision values. Through the clinical application of this automatic model, temporal analysis of VFSS will be easier and more accurate.

## Introduction

Dysphagia, defined as difficulty in swallowing, is caused by neurological, muscular, anatomical, or psychological factors^1–3^. As dysphagia can lead to serious complications such as malnutrition, dehydration, aspiration pneumonia, and choking, accurate diagnosis is crucial^4–6^.

Several diagnostic methods are used to diagnose dysphagia. Videofluoroscopic swallowing studies (VFSS) and fiberoptic endoscopic evaluation of swallowing (FEES) are the gold standards for dysphagia evaluation^7^. Among these, VFSS is commonly used for dysphagia assessment because it provides functional as well as structural information^8,9^. A VFSS is a dynamic, continuous radiological examination of swallowing. Dynamic videos of the relevant anatomic structures (generally lateral and frontal views of the oral cavity, pharynx, and upper esophagus) during swallowing with various volumes and viscosities of the contrast-mixed bolus are obtained. By analyzing these videos, the examiner can not only detect the type and severity of dysphagia, but also suggest postural maneuvers or therapeutic interventions^10,11^. However, depending on the subjective visuoperception of the examiner, adequate intra- and inter-rater reliability cannot always be ensured^12,13^. Hence, various tools and sequential temporal analysis for VFSS are used for both clinical and research purposes to achieve a more objective and quantitative evaluation^14–18^. Quantitative temporal measurements of swallowing events can provide information regarding swallowing dynamics and the coordination of swallowing events. Specific swallow event delays were correlated with specific disabilities or stroke lesions in patients. Patients with decreased cognitive function commonly show oral phase delay, and those with brainstem stroke may show prolonged upper esophageal opening duration^19–21^. If there is a noticeable delay in a specific swallowing event, it may be used to predict or suspect a specific disease.

However, manual analysis can be time consuming and complicated for clinicians^22^. Hence, studies have been conducted using deep learning to analyze VFSS videos^22–26^. For the spatial analysis, two studies used deep learning to automatically detect hyoid bone movement and airway invasion^22,26^. Previous studies have applied deep learning for the temporal analysis of VFSS. Most of these studies focused on detecting one specific phase, such as the pharyngeal phase or pharyngeal delay time^23–25^. However, all the other phases also have clinical significance; thus, automating the temporal analysis of the entire swallowing process would be more valuable in clinical aspects compared to the previous models. Moreover, as more time is consumed in manually analyzing entire phases than only a few phases such as the pharyngeal phase or pharyngeal delay time, invention of a deep learning model detecting the whole phase of the swallowing process will ultimately result in a more significant reduction in time and costs. If automatically detecting and quantifying the lengths of all swallowing phases are possible, clinicians may quickly and precisely identify the problematic phase. Moreover, clinicians may easily detect changes of patients swallowing function overtime.

Therefore, the purpose of this study was to develop an automatic model to measuring seven distinct temporal parameters of the VFSS during the overall swallowing process. Our approach of simultaneously detecting multiple phases differs from the previous works which primarily concentrated on detecting one specific phase^23–25,27^. In this study, we proposed a novel deep learning model for temporal localization of swallowing phases. We evaluated the performance of the proposed model on our VFSS dataset in comparison with traditional models for deep learning.

## Methods

### Subjects

Patients who complained of dysphagia or were suspected of having dysphagia underwent VFSS at Korea University Guro Hospital between September 2020 and September 2021 and were consecutively recruited for this study. Overall, 594 VFSS videos from 462 patients were retrospectively reviewed. The exclusion criteria were as follows: (1) age ≤ 19 years (2) inability to progress from the pharyngeal phase to the esophageal phase because full analysis of the temporal parameters in these patients was not possible (3) incomplete recording of videos and (4) insufficient or low contrast of the video precluding identification of anatomic structures. Based on the exclusion criteria, 47 videos from 39 patients were excluded. Consequently, 547 videos of 423 patients were included as ground-truth data samples. This study was approved by the Institutional Review Board of the Korea University Guro Hospital (IRB No. 2021GR0568) and the institutional review board waived the requirement to obtain the informed consent. This study was performed in accordance with the guidelines of the Declaration of Helsinki.

### VFSS analysis

The VFSS was performed by a single physician. The participants were seated in an upright position and swallowed barium-mixed materials. A lateral view of the head and neck region was recorded at a frequency of 15 frames per second (FPS) using a Sonialvision G4 radio-fluoroscopy system (Shimadzu Medical Systems and Equipment, Japan). Various amounts and viscosities of materials were used for the VFSS examination, including thin liquid 2 cc, thin liquid 5 cc, thin liquid with a cup, semi-liquid 2 cc, semi-liquid 5 cc, semi-solid, and solid materials. Each material was tested at least twice. All the materials were mixed with barium powder. Among them, the thin liquid was then mixed with barium powder as about 35% w/v. Only videos of swallowing 2 cc of thin liquid were included in this study since our hospital protocol initiates the examination with thin liquid 2 cc as the first material resulting in no residual barium in the oropharyngeal area at the beginning of the videos, leading to easier analysis, Two experienced physicians independently analyzed the VFSS video clips. One physician had  ≥ 15 years of experience in VFSS analysis, while the other one had  ≥ 3 years of experience. The intraclass coefficient was 0.999 (*p*-value  < 0.001). If any disagreements occur between the two clinicians, a consensus was reached through discussion. In our hospital’s routine protocol, VFSS video analysis is conducted not only using the Penetration-Aspiration Scale^28^ but also qualitatively evaluating other several items visuoperceptually. The items included here are similar with the modified barium swallowing study (MBSS) tool (MBSImp)^29^. For this research, we applied the ASPEKT method, particularly from step 1 to 4, for our video preparation and analysis^14^ as follows: (1) extraction of the first subswallow video, (2) major event labeling, and (3) temporal parameter measurement.

### Extracting the first sub-swallow video

If multiple sub-swallows were present, only the first sub-swallow video was extracted. We set the first frame as the starting point of the oral phase, and the last frame as the point between the swallow rest and the start of the next sub-swallow.

### Major event labeling

**Figure 1:**
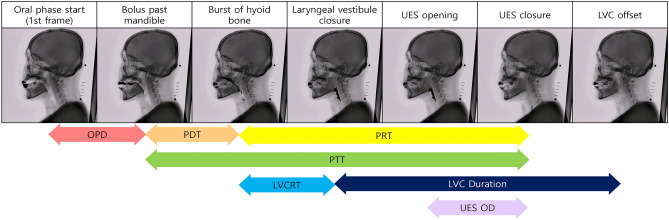
Phase description. LVC laryngeal vestibule closure, UES upper esophageal sphincte. Oral phase duration is the interval between oral phase start and bolus passing through the mandible. Pharyngeal delay time means interval between bolus passing through the mandible. Pharygeal response time is the interval between the burst of hyoid bone and UES closure. Pharyngeal transit time is interval between bolus passing through the mandible and UES closure. LVC reaction time is the interval between burst of the hyoid bone, LVC duration is the interval between laryngeal vestibule closure. UES opening duration is the interval between UES opening and UES closure.

As presented in Fig. 1, major event labeling was performed similar to that in the ASPEKT method devised by Steele et al.^14^. The adjusted definitions of the major events are as follows:

#### Start of oral phase

This was defined as the time at which the bolus first entered the oral cavity. It was also the first frame of the video clip used for temporal analysis.

#### Bolus past mandible

This is the first frame in which the leading edge of the bolus touches or crosses the ramus of the mandible. If the two mandibular lines did not overlap, the midpoint between the upper and lower mandibular lines was considered the reference point.

#### Burst of hyoid bone

This is the first frame in which the hyoid bone starts jumping anterosuperiorly.

#### Laryngeal vestibule closure (LVC)

This event is the first frame when the inferior surface of the epiglottis and arytenoid process contact. When LVC occurs, the air space in the laryngeal vestibule becomes invisible. When LVC occurs incompletely, the frame in which the arytenoid process and inferior surface of the epiglottis are most approximate is used.

#### Upper esophageal sphincter (UES) opening

The UES first opens as a bolus or air passes through it.

#### UES closure

This is the first frame in which a single point or part of the UES closes behind the tail of the bolus.

#### LVC offset

This is the earliest frame in which the air space in the laryngeal vestibule becomes visible.

### Temporal parameter measurement

Seven temporal parameters including oral phase duration, pharyngeal delay time, pharyngeal response time, pharyngeal transit time, laryngeal vestibule closure reaction time, laryngeal vestibule closure duration and upper esophageal sphincter opening duration were measured using labeled major events, similar to the ASPEKT method devised by Steele et al.^14^. (Fig. 1) As fluoroscopy was projected at 15 FPS, using the frame number of the main events, the following temporal parameters could be calculated in milliseconds.

### Development of automatic models

**Figure 2:**
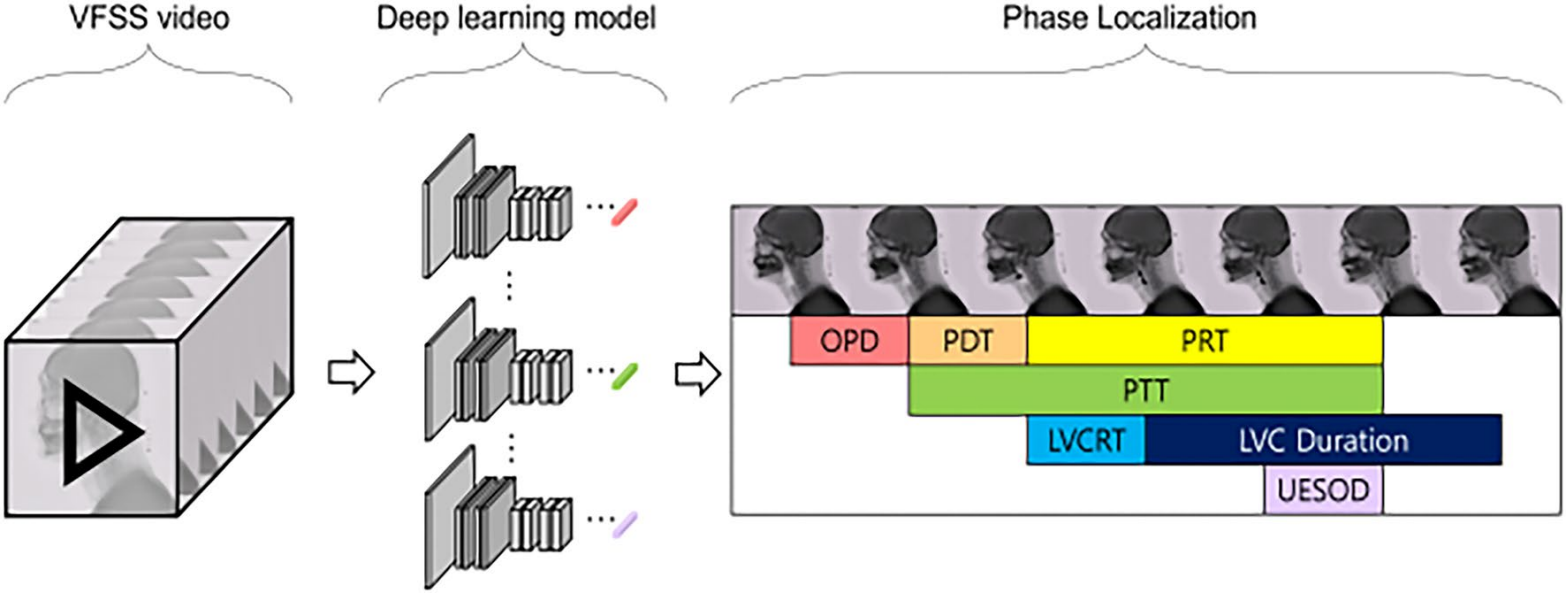
Conceptual diagram of the proposed framework. OPD: oral phase duration. PDT: pharyngeal delay time. PRT: pharyngeal response time. PTT: pharyngeal transit time. LVCRT: laryngeal vestibule closure reaction time. LVC Duration: laryngeal vestibule closure duration. UESOD: upper esophageal sphincter opening duration.

Figure 2 presents an overview of the proposed method for phase localization using deep learning.

### Three-dimensional convolutional neural network (3D-CNN)

A CNN is a type of artificial neural network based on convolution operations. The convolution operation effectively extracts and combines the local image features and is applied to the input image multiple times, each of which is called a convolutional layer. Two-dimensional (2D) CNNs are typically used to analyze 2D images in deep learning. To process video data, 3D-CNNs can be applied, which add a time dimension to 2D images for input processing, that is, the video is processed as a temporal sequence of images. In this study, we adopted ResNet3D, a type of 3D-CNN^30^.

### Models

**Figure 3:**
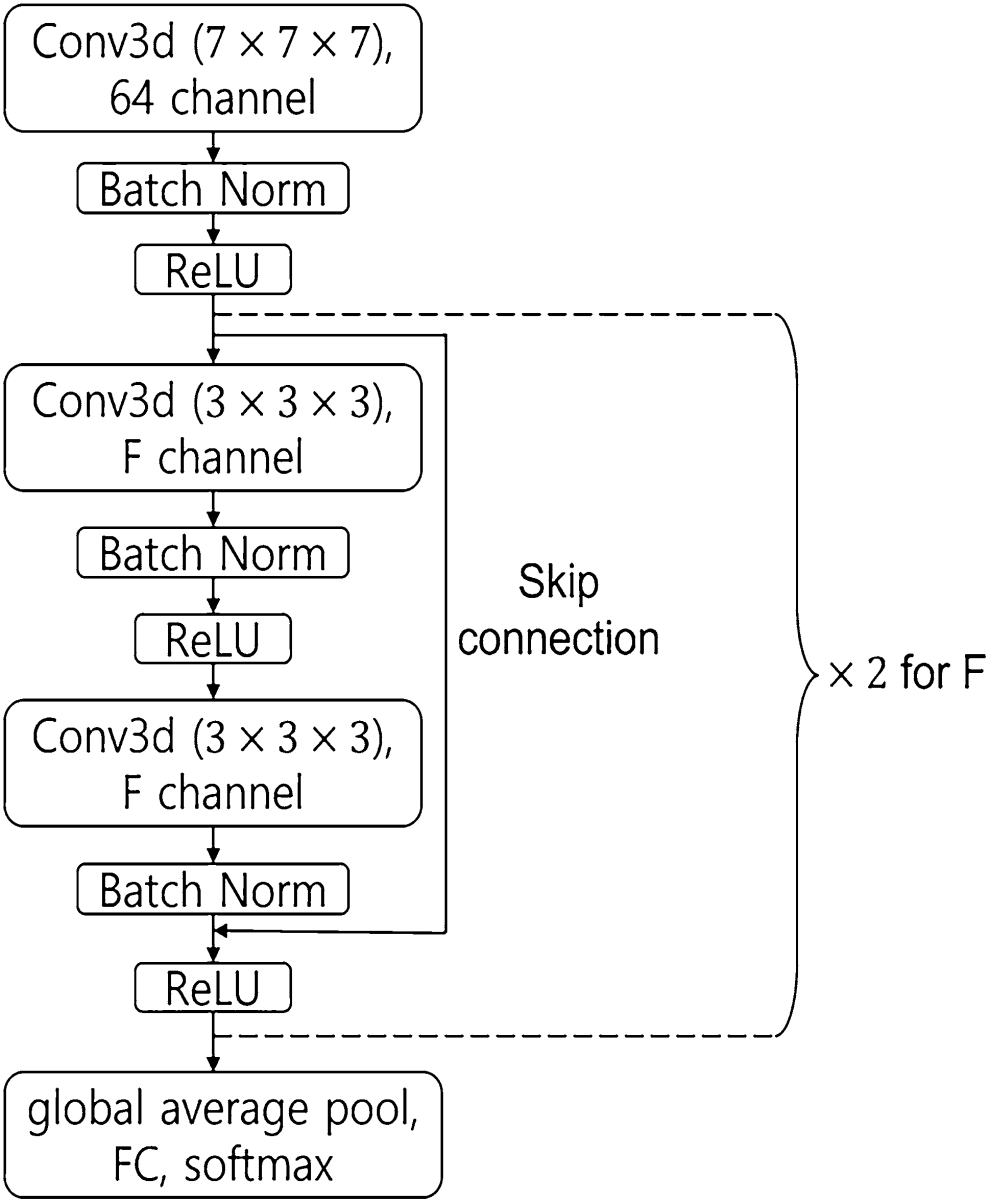
ResNet3D structure. F = [64, 128, 256, 512]. Conv3d represents the 3D convolution layer and the numbers in parentheses denote the kernel dimensions of the convolutional filter. Batch normalization (Batch Norm) stabilizes the training process. ReLU (Rectified Linear Unit) is an activation operation that allows model to learn non-linearities to enhance the expressivity of the model. The global average pool summarizes features. Fully Connected (FC) layer and softmax function yield the class probability.

The base architecture used was ResNet3D-18^30^. It has skip connections in which the input bypasses the intermediate layers and is fed directly to the output. Skip connections facilitate deep neural network trainings. The model comprised multiple residual blocks containing several convolutional layers and one skip connection. The residual block is illustrated in Fig. 3. We adopted three architectural variants of ResNet3D-18, which are described as follows:

#### DEFAULT

This variant uses the default configuration of ResNet3D-18 with changes only in the number of input frames. In this study, we conducted experiments using input frame lengths of 7 or 13. The input label was set at the center of the input window. For example, the label of the input of seven frames is set to 1 if the fourth frame of the input is in the phase of interest, and 0 otherwise.

#### BIDIRECTIONAL

**Figure 4:**
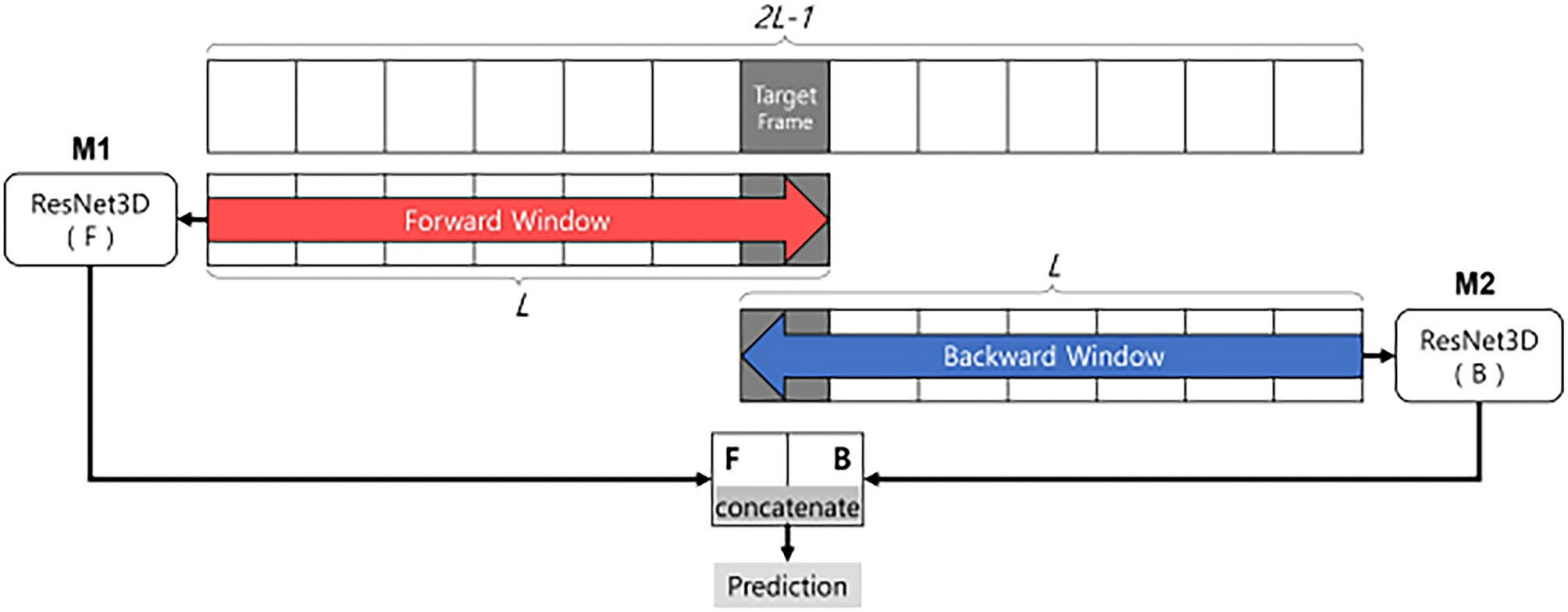
Bidirectional variant of ResNet3D.

We proposed a new architectural variant of ResNet3D. Inspired by bidirectional recurrent neural networks, we introduced a bidirectional structure in ResNet3D that captures the forward and backward stream features of a target frame as follows^31^ (Fig. 4).

The bidirectional model combines the predictions from two separate ResNet3D models. Specifically, we used two models, denoted by $\mathbf{M}1$ and $\mathbf{M}2$, each of which takes $L$ frames as input, aiming to predict the label of the center frame of $2L-1$ frames. The input for $\mathbf{M}1$ is the first $L$ successive frames, that is, from the first frame to the center of the loaded frames. The input for $\mathbf{M}2$ is the last $L$ successive frames, that is, from the center of the loaded frames to the last frame. Thus, $\mathbf{M}1$ should learn forward temporal information, and $\mathbf{M}2$ should learn backward temporal information relative to the center frame. The outputs from $\mathbf{M}1$ and $\mathbf{M}2$ are combined and concatenated, and the combined vector was passed to a fully connected layer to yield the final prediction output.

#### CONV-SA

**Figure 5:**
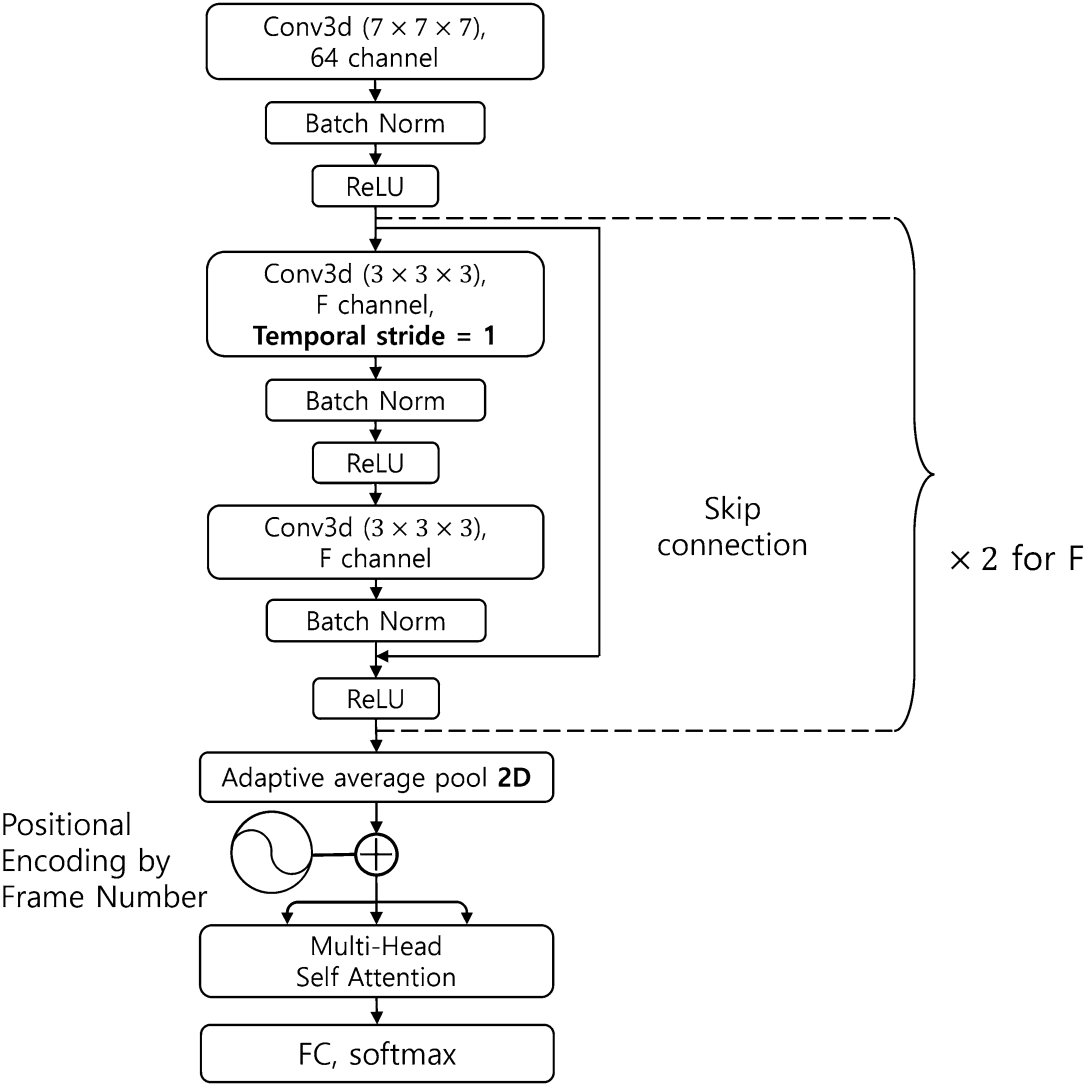
CONV-SA variant of ResNet3D.

We proposed another novel architectural variant for frame-level temporal action localization in videos (Fig. 5). This architecture predicts a label for each frame within a video segment by capturing inter-frame dependencies using self-attention layer from the Transformer^32^ architecture. To achieve this, the default convolution stride configuration of ResNet3D-18 is fixed to 1. This makes the temporal sequence length of both the input and output of ResNet3D-18 the same. To capture the serial relations among the frames, the frame number information is added to the outputs from ResNet3D-18 as like positional encoding implemented in the Transformer architecture. Subsequently, the incorporated features are fed into a multihead attention layer and a Fully Connected (FC) layer to yield the final prediction output. This architectural variant is dubbed “CONV-SA” which stands for convolution-self-attention.

### Experimental settings

#### Datasets

The total number of VFSS videos was 547. The video dataset was split into training, validation, and test sets with 444, 49, and 54 videos, respectively. The model input was red, green, and blue (RGB) video frames resized to 224 × 224 pixels. from 1024 × 1024 pixels to reduce computational complexity while preserving information essential for the phase detection.

#### Baselines

We compared our model with those of prior studies on VFSS. Lee et al. proposed a model based on VGG-16, a neural network with 16 weight layers that is widely used for image recognition^24,33^. The I3D described by Lee et al. is a network that has been widely applied to action recognition and classification tasks^25,34,35^. We trained the baseline models on our dataset and optimized the hyperparameters.

#### Transfer learning

We adopted the following transfer learning technique. Deep learning models must be trained using a large amount of data. However, collecting large-scale medical data is difficult because of privacy issues or the small number of participants. Transfer learning retrains a model pretrained on large datasets of generic images or videos. All models considered in this study used transfer learning. For example, VGG-16 was pretrained on ImageNet which contains 200 categories of 14,197,122 images^33,34,36^. The I3D was pretrained on a Kinetics-600^34,37^. The ResNet3D variants used in this study were pretrained using Kinetics-700^38^. The Kinetics datasets contained 650,000 human action video clips. Each clip was annotated with a single action class and lasted approximately 10 s. Kinetics-600 and Kinetics-700 contain videos of 600 and 700 action classes, respectively. The pretrained model yielded a substantially higher performance than those that were not pretrained.

#### Performance evaluation

The accuracy, F1 score, and average precision (AP) were calculated to comprehensively evaluate the temporal localization of the phases in the test dataset.

$${\text{accuracy}}= \frac{TP+TN}{TP+TN+FN+FP}$$

$${\text{recall}}= \frac{TP}{TP+FN}$$

$${\text{precision}}= \frac{TP}{TP+FP}$$

$$\text{F1 score}= \frac{2}{\frac{1}{\text{recall}}+\frac{1}{\text{precision}}}= \frac{TP}{TP+\frac{1}{2}(FP+FN)}$$

TP stands for the number of true-positive frames. In other words, it is the number of frames which belong to a phase and are classified to be that phase by our model. FP (false positive), TN (true negative), and FN (false negative) can be defined in a similar manner. AP is the area under the precision-recall graph, with an upper bound of 1 and higher values indicating better results. AP is a widely used metric for the temporal action localization task in computer vision and is particularly relevant to cases with class imbalance, that is, if only a small fraction of the given frames is the phase to be detected, as was in our case.

### Ethics approval and consent to participate

This retrospective study was approved by the Korea University Guro Hospital Institutional Review Board (IRB No. 2021GR0568).

## Results

Of the 594 videos from 462 participants, 47 were excluded based on the exclusion criteria. Finally, 547 subjects (71.8 ± 12.7 years; 331 men and 216 women) were included to develop the automatic models. Stroke is the most common cause of dysphagia. The causes of the dysphagia and penetration and aspiration score are described in Table 1^28^.

**Table 1:** Causes of dysphagia and penetration and aspiration scale.

Cause of dysphagia	
Stroke	207
Brain tumor	21
Traumatic brain injury	20
Parkinson’s disease	17
Cervical myelopathy	14
Head and neck cancer	12
Guillain–Barre syndrome	10
Encephalitis	9
Epilepsy	8
Esophageal cancer	7
Dementia	6
Motor neuron diseases	4
Myopathy	4
Cranial nerve lesion	3
Neuromuscular junctional disorder	1
Other medical condition	191
Unknown	13
Penetration Aspiration Scale	
1	406
2	31
3	45
4	2
5	10
6	11
7	8
8	34

Values are number of patients.

The temporal parameters and numbers of frame corresponding to each swallowing phase are summarized in Table 2.

**Table 2:** Swallowing phase durations and number of frames.

Phase	Phase duration (s)	Number of Frames
Oral phase duration	1.844 ± 2.281	27.658 ± 34.215
Pharyngeal delay time	1.387 ± 2.857	20.809 ± 42.856
Pharyngeal response time	0.677 ± 0.137	10.148 ± 2.060
Pharyngeal transit time	1.978 ± 2.880	29.676 ± 43.195
LVC reaction time	0.302 ± 0.112	4.526 ± 1.687
LVC duration	0.676 ± 0.266	10.053 ± 3.987
UES opening duration	0.432 ± 0.116	6.483 ± 1.746

Values are mean ± standard deviation.

*s* Second, *LVC* Laryngeal vestibule closure; *UES* Upper esophageal sphincter.

We repeated the experiments for all phases 20 times for each model, averaged the results, and rounded the numbers to three decimal places. For three metrics, the ResNet3D variants outperformed all the baseline models in every phase of swallowing (Table 3). We conducted Tukey^39^ test to see if the outperformance of our model over baseline methods was statistically significant with a significance level of 0.05. The analysis revealed that it was statistically significant that the proposed model outperformed the VGG model in all the phases (p value was at most $7.6\times {10}^{-6}$). Meanwhile, it was statistically significant that the proposed model outperformed I3D model in the case of four phases: Oral Phase Duration (*p* = $9.4\times {10}^{-16}$), Pharyngeal Delay Time (*p* = $3.2\times {10}^{-8}$), Pharyngeal Transit Time (*p* = $3.2\times {10}^{-12}$), UES opening duration (*p* = 0.00191), but not for three phases: Pharyngeal Response Time (*p* = 0.03898), LVC Reaction Time (*p* = 0.05762), LVC Duration (*p* = 0.05382). The architectural variants with the highest performance in each swallowing phase varied, as listed in Table 4. The configuration of window sizes for both the DEFAULT and BIDIRECTIONAL variants was seven frames, while that for the CONV-SA variant was fifty-frames. In the multi-head attention layer of CONV-SA, we used four heads.

**Table 3:** Performance results of models.

Model	Metric		
	Accuracy	F1 score	Average precision
Oral phase duration			
VGG	0.898 ± 0.010	0.832 ± 0.016	0.729 ± 0.022
I3D	0.918 ± 0.014	0.866 ± 0.020	0.775 ± 0.031
Proposed	0.966 ± 0.008*	0.941 ± 0.013*	0.899 ± 0.023*
Pharyngeal delay time			
VGG	0.837 ± 0.033	0.644 ± 0.158	0.572 ± 0.079
I3D	0.861 ± 0.008	0.697 ± 0.025	0.633 ± 0.021
Proposed	0.901 ± 0.024*	0.794 ± 0.062*	0.737 ± 0.062*
Pharyngeal reaction time			
VGG	0.94 ± 0.007	0.817 ± 0.015	0.687 ± 0.022
I3D	0.976 ± 0.004	0.915 ± 0.013	0.853 ± 0.022
Proposed	0.978 ±0.002*	0.924 ± 0.007*	0.865 ± 0.012*
Pharyngeal transit time			
VGG	0.858 ± 0.009	0.809 ± 0.017	0.766 ± 0.012
I3D	0.866 ± 0.010	0.813 ± 0.018	0.786 ± 0.013*
Proposed	0.905 ± 0.015*	0.873 ± 0.023*	0.846 ± 0.02*
LVC reaction time			
VGG	0.946 ± 0.013	0.668 ± 0.042	0.48 ± 0.043
I3D	0.979 ± 0.003	0.818 ± 0.043	0.688 ± 0.052
Proposed	0.98 ± 0.003*	0.837 ± 0.031*	0.714 ± 0.041*
LVC duration			
VGG	0.957 ± 0.005	0.859 ± 0.012	0.752 ± 0.019
I3D	0.963 ± 0.005	0.867 ± 0.014	0.769 ± 0.023
Proposed	0.966 ± 0.007*	0.875 ± 0.035*	0.787 ± 0.041*
UES opening duration			
VGG	0.965 ± 0.008	0.844 ± 0.028	0.727 ± 0.042
I3D	0.976 ± 0.007	0.868 ± 0.055	0.779 ± 0.065
Proposed	0.981 ± 0.002*	0.905 ± 0.01*	0.830 ± 0.017*

Values are presented as means ± standard deviations.

*LVC* Laryngeal vestibule closure; *UES* Upper esophageal sphincter.

*Best result among the three models.

**Table 4:** ResNet3D architectural variants of the best performance for each phase.

Phase	Variants
Oral phase duration	CONV-SA
Pharyngeal delay time	CONV-SA
Pharyngeal response time	DEFAULT
Pharyngeal transit time	CONV-SA
LVC reaction time	BIDIRECTIONAL
LVC duration	BIDIRECTIONAL
UES opening duration	CONV-SA

*LVC* Laryngeal vestibule closure; *UES* Upper esophageal sphincter.

## Discussion

Our study successfully proposed an automatic model that can perform temporal analysis of the entire swallowing phase using VFSS data. This is particularly valuable because it can be easily applied in clinical practice as the overall swallowing phase was analyzed using realistic datasets without special manipulation.

Although some studies using deep learning for the temporal analysis of VFSS have been presented, most of them focused on detecting a specific phase, such as the pharyngeal phase or pharyngeal delay time^23–25^. To the best of our knowledge, this is the first study to measure various temporal parameters throughout the swallowing process using deep learning. In addition, deep learning models previously used for the automated temporal analysis of VFSS have limitations. A study that used the VGG model^24^, which is a 2D-CNN, did not consider the temporal relationships of the frames; thus, it may not learn the important muscle movements that determine the phase or event. In contrast, our study used 3D convolution operations to capture the temporal relationships between frames. Another study that used an I3D model^25^ did not classify whether a frame belonged to the phase of interest, but instead it classified whether the input frames overlapped with the phase of interest. However, our proposed method can classify each frame by detecting the presence of a phase at the center of the input frame. Lee et al.^23^ proposed a model which used three stages for phase detection, required training over optical flow data in addition to RGB data. Our method uses only RGB data and consists of only one stage, which lowers the computational cost. Bandini et al.^27^ proposed to detect only pharyngeal phase using existing models, and the requirement was that the phase must be longer than three frames. By contrast, we proposed a novel deep learning model which yields the best performance for phase detection which works even when the phase is shorter than three frames.

**Figure 6:**
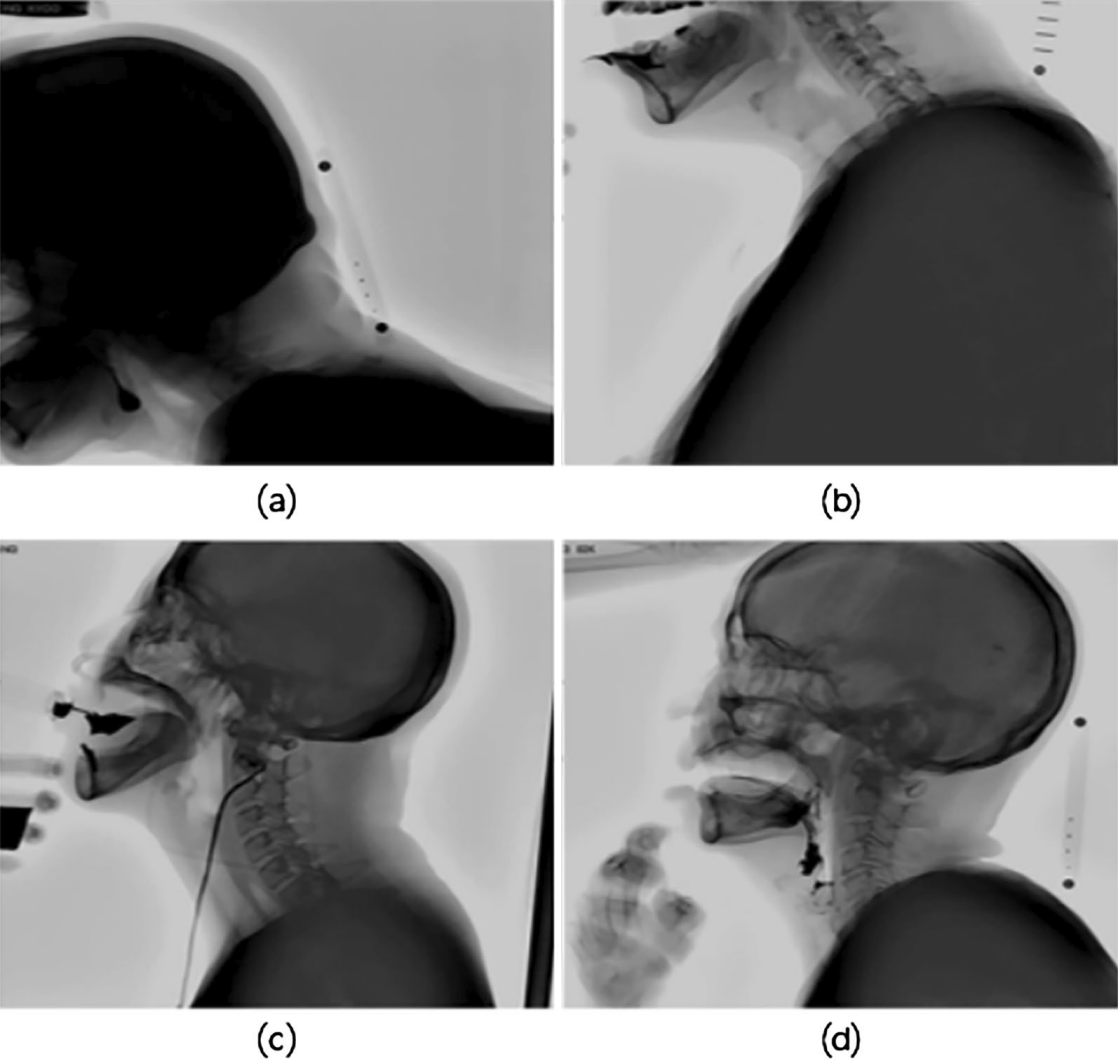
Example of irregularities in our video data. (**a**) and (**b**) The patient is out of the screen owing to the inability to maintain posture. (**c**) The hand of a clinician, apparatus, and an obstacle over the throat were captured in the video, (**d**) the patient’s hand was captured in the video.

Our study was conducted using the VFSS videos of patients examined in clinical practice. In these videos, the head and shoulders of the patients, as well as the oral and pharyngeal areas were included. During VFSS, the head and shoulders of the patients may assume various positions and sometimes move back and forth. Occasionally, the hands of a clinician or patient may have appeared. Moreover, some videos had low clarity because of poor focus or contrast (Fig. 6). In reality, some patients may have difficulty in maintaining proper posture or consistently maintaining a fixed posture. In fact, the performance of the baseline models reproduced using our data was lower than that reported in the original papers^24–26^ (Table 3). This was because the models were trained using different datasets. The anatomical extent recorded in the video clips used in previous studies was limited to the area around the neck; thus, training or evaluating the model using realistic datasets for its applicability in clinical practice is important.

Among these phases, the performance of the model detecting the pharyngeal delay time and LVC reaction times yielded relatively poor results compared to another phase. These phases are different from others as the terminal point of the phases sometimes precedes the initiation point. For example, in the pharyngeal delay time, the action of the burst of the hyoid bone event typically follows the event of a bolus passing the mandible, although sometimes the burst of the hyoid bone event precedes the bolus passing the mandible event. This made training of the model more difficult.

We conducted t-test with a significance level of 0.05 in terms of classification performance based on gender, age (greater than 65 or otherwise), Penetration-Aspiration Score (greater than 5 or otherwise). Across these variables, our findings revealed no significant differences in the performance of the proposed models in all the phases except for detecting LVC Reaction time. In this case, when the data was categorized based on Penetration-Aspiration Score (PAS), the two-tailed p-value was 0.04, indicating a statistically significant difference in classification performances. The performance for the group with a lower PAS was better than the one with PAS greater than 5.

This study had some limitations. First, unlike many previous studies that used 30 FPS machines, we used a fluoroscopy machine with 15 FPS. Although this allowed us to reduce the radiation dose to the patients, the results of the temporal analysis may have been less accurate. Second, the participants who could not progress from the pharyngeal to the esophageal phase because of severe UES dysfunction were excluded from this study; therefore, analyzing these cases using this model would be difficult. Third, only videos in which patients swallowed 2 cc of liquid were analyzed. No residue in the videos was noted since 2 cc of the thin liquid was the first to be examined among the materials of various consistencies and volumes. Therefore, only videos of patients swallowing a 2 cc thin liquid were used to develop a more accurate model. The accuracy of the model can be improved if further studies include cases where previous residues remain, as well as using various volumes and viscosities of the materials.

In future work using deep learning models for VFSS, we plan to develop models for multi-labeled event detection instead of phase localization. Event detection indicated that the model detected important changes in anatomical structures that determined the phases of the swallowing process. For example, if a model could detect the events of a bolus past the mandible and a burst of the hyoid bone, identifying the pharyngeal delay time could be easier. This is because detecting individual events involving movements is easier than detecting the entire phase. Additionally, in order to strengthen the rationale for utilizing deep learning in VFSS video analysis, we are planning to conduct a future study quantitatively comparing the time-saving potential of deep learning techniques in comparison to manual analysis. Moreover, in the actual diagnostic process of dysphagia, there are various components involved not only temporal analysis but also PAS rating and the elements encompassed within the MBSImP, etc. Therefore, our future objective is to automate all of these components and quantify the extent to which deep learning can save time compared to manual analysis in the diagnosis of dysphagia in VFSS.

## Conclusion

We developed an automatic model that included various temporal parameters of the VFSS. The proposed ResNet3D variants outperformed existing models in detecting all phases of the swallowing process. This automatic model reduces clinician labor and allows faster assessment of dysphagia.

## Data availability

The datasets for this study are available from the corresponding author on reasonable request.

## Abbreviations

VFSS: Videofluoroscopic swallowing study
FEES: Fiberoptic endoscopic evaluation of swallowing
LVC: Laryngeal vestibule closure
UES: Upper esophageal sphincter
FPS: Frames per second
3D: Three-dimensional
CNN: Convolutional neural network
RGB: Red, green and blue
AP: Average precision
TP: True positive
PAS: Penetration-aspiration score
