# Author Correction: Application of deep learning technology for temporal analysis of videofluoroscopic swallowing studies

**DOI:** 10.1038/s41598-024-52899-3

**Published:** 2024-01-30

**Authors:** Seong Yun Jeong, Jeong Min Kim, Ji Eun Park, Seung Jun Baek, Seung Nam Yang

**Affiliations:** 1 Department of Computer Science and Engineering, Korea University, 145 Anam-ro Seongbuk-gu, Seoul, 02841 Korea; 2 Department of Physical Medicine and Rehabilitation, Korea University Guro Hospital, Korea University College of Medicine, 148, Gurodong-ro, Guro-gu, Seoul, 08308 Korea

Correction to: *Scientific Reports* 10.1038/s41598-023-44802-3, published online 16 October 2023

The Funding section in the original version of this Article was incomplete.

“This research was supported by the MSIT (Ministry of Science and ICT), Korea, under the ICT Creative Consilience program (IITP-2022-2020-0-01819) supervised by the IITP (Institute for Information & communications Technology Planning & Evaluation).”

now reads

“This research was supported by the MSIT (Ministry of Science and ICT), Korea, under the ICT Creative Consilience program (IITP-2022-2020-0-01819) supervised by the IITP (Institute for Information & communications Technology Planning & Evaluation), and by the National Research Foundation of Korea (NRF) Grant through MSIT under Grant No. 2022R1A5A1027646 and No. 2021R1A2C1007215.”

The original Article has been corrected.

